# Design of Preamplifier for Ultrasound Transducers

**DOI:** 10.3390/s24030786

**Published:** 2024-01-25

**Authors:** Hojong Choi

**Affiliations:** Department of Electronic Engineering, Gachon University, 1342 Seongnam-daero, Sujeong-gu, Seongnam 13120, Republic of Korea; hojongch@gachon.ac.kr; Tel.: +82-31-750-5591

**Keywords:** preamplifier, ultrasound transducer, ultrasound system

## Abstract

In diagnostic ultrasound imaging applications, preamplifiers are used as first-stage analog front-end amplifiers for ultrasound transducers because they can amplify weak acoustic signals generated directly by ultrasound transducers. For emerging diagnostic ultrasound imaging applications, different types of preamplifiers with specific design parameters and circuit topologies have been developed, depending on the types of the ultrasound transducer. In particular, the design parameters of the preamplifier, such as the gain, bandwidth, input- or output-referred noise components, and power consumption, have a tradeoff relationship. Guidelines on the detailed design concept, design parameters, and specific circuit design techniques of the preamplifier used for ultrasound transducers are outlined in this paper, aiming to help circuit designers and academic researchers optimize the performance of ultrasound transducers used in the diagnostic ultrasound imaging applications for research directions.

## 1. Introduction

Ultrasound systems are widely used in medical, nondestructive, photoacoustic, and stimulation applications [1,2,3]. Recently, ultrasound systems have garnered increasing attention owing to new technologies such as capacitive micromachined ultrasonic transducers, asynchronous specific integrated circuit (ASIC) fabrication processes, smartphone-based ultrasound machines, photoacoustic imaging, and magnetic resonance-guided brain stimulation [4,5,6,7].

**Figure 1:**
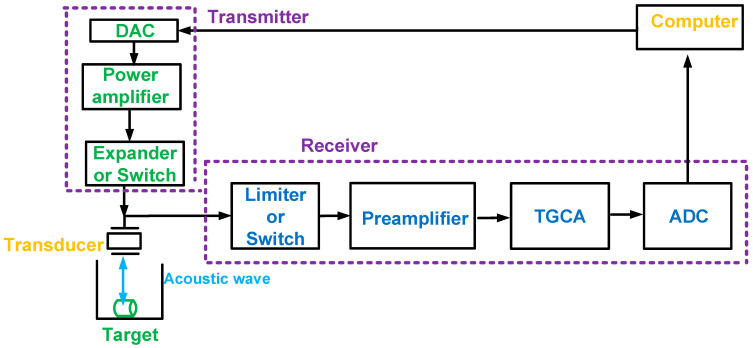
Block diagram of the transducer and ultrasound transmitter and receiver used for diagnostic ultrasound imaging applications.

In diagnostic ultrasound imaging applications, the ultrasound systems are categorized into transmitters, receivers, and transducers [8,9,10]. Figure 1 shows the transducer, transmitter, and receiver in the ultrasound system used to describe the locations of the components of the preamplifier and time-gain compensation amplifier [11,12]. The computer-controlled digital-to-analog converter (DAC) produces low-voltage single or multiple-cycle pulse signals [13,14]. High-voltage pulse signals, amplified by the power amplifier in the transmitter, trigger the transducer through an expander or switch [15]. A limiter or switch protects the receiver from high-voltage or high-power signals generated by the power amplifiers because of the shared path between the transmitter and receiver [16].

The preamplifier is one of the first-stage receiver electronic devices after the transducer that amplifies weak acoustic signals with fewer noise effects [17]. Considering a transducer with low sensitivity requires a high input dynamic range of the preamplifier, the preamplifier used for ultrasound applications is a Class-A-type amplifier that continuously conducts voltage and current [18]. This preamplifier operates continuously during pulse transmission and echo reception; therefore, switches are utilized to block unwanted pulse signals and reduce power consumption; switches in the IC are normally implemented using voltage-controlled metal-oxide-semiconductor field-effect transistor (MOSFET) switches to save occupied chip space [19,20]. The low-voltage, current, or power signals received from the transducer are amplified by the preamplifier and time-gain-compensation amplifier (TGCA) in the receiver and then digitized with an analog-to-digital converter (ADC) to obtain the images [21]. The TGCA needs to amplify the weak signals further when the attenuation of the ultrasound signals is exponentially degraded, depending on the target distance [22]. In Figure 1, the transmitting and receiving beamforming components of the ultrasound transducer array are excluded to simplify the description of the entire ultrasound system. In a diagnostic photoacoustic system, the transmitter side is replaced by light-generating sources such as lasers, light-emitting diodes, or radio frequency sources [23,24,25].

The output of the capacitive micromachined ultrasonic transducer (CMUT) device is current; thus, a transimpedance amplifier is used to convert the current generated from the CMUT in the input to voltage in the preamplifier output [26]. Therefore, the preamplifiers were designed as voltage and current (transimpedance) amplifiers for the piezoelectric transducer and CMUT, respectively. The output of the piezoelectric transducer is a voltage; therefore, a low-noise voltage operational amplifier was used [27]. The preamplifier, also known as a low-noise amplifier (LNA), is used in piezoelectric transducers [26].

Section 2 describes the design parameters of the preamplifiers, such as voltage or current gain, bandwidth, direct current (DC) power consumption, and input- or output-referred noises, or noise figures. Section 3 presents the topology, design parameters, and circuit design techniques of previously reported preamplifiers for specific diagnostic ultrasound imaging applications such as CMUT, piezoelectric transducer, and imaging. Section 4 discusses the design topologies and criteria for the currently developed preamplifiers used for diagnostic ultrasound imaging applications and summarizes this review.

## 2. Design Parameters of the Preamplifiers for Ultrasound Transducer Types

**Figure 2:**
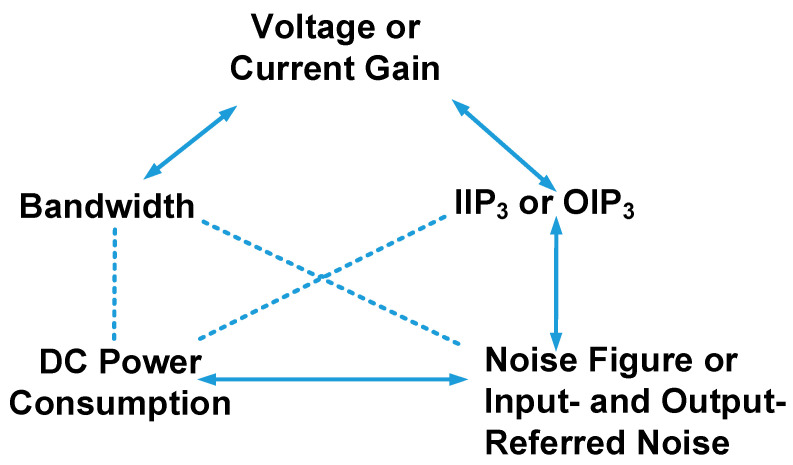
Design parameters of the preamplifiers used for ultrasound transducers.

The design parameters of preamplifiers for ultrasound transducer types are described in this section. Figure 2 shows the relationship between the design parameters of the preamplifiers used for diagnostic ultrasound imaging applications because design engineers for ultrasound components or systems need to consider the trade-off relationship at the design level. The design parameters of the preamplifiers were based on information from several textbooks on analog circuits, ICs, amplifiers, and ultrasound systems [28,29,30,31,32,33]. These design parameters are useful for circuit design engineers because some ultrasound systems require specific performance parameters.

The gain of the preamplifier is an important parameter because the weak echo signal generated by the transducer must be amplified. The voltage or current gain parameters are the extent to which the input signals are amplified [34]. Owing to the limited space for intravascular ultrasound (IVUS) applications, most research has focused on developing capacitive micromachined ultrasonic transducer devices with integrated circuits (IC) closely attached between the CMUT and IC [35]. For IVUS areas, the small size ultrasound transducers are required due to limited areas so the received echo signals are very weak so the high gain of the preamplifier is preferable. The bandwidth of the preamplifier is typically at least twice or higher than that of the transducer because the harmonic imaging mode requires the use of second or higher-order harmonic components to improve the image resolution [36]. The bandwidth can be increased while the gain reduces if the preamplifier has an operational amplifier topology [37]. A preamplifier design with a high gain has high power consumption because a high gain requires a high biasing current in the preamplifier [38,39]. While a preamplifier with high linearity is desirable for producing extremely weak acoustic signals from transducers, these signals affect the maximum gain performance of the amplifier.

The input third-order intercept point (IIP_3_) or the output third-order intercept point (OIP_3_) is the intercept point at which the component at the fundamental frequency and third-order intermodulation distortion points meet [40]. They are useful parameters to show the linearity of the preamplifiers. The higher the IIP_3_ or OIP_3_, the more linear the preamplifier works. Therefore, the circuit designers can increase the voltage gain before the intermodulation distortion is started [40]. In the harmonic imaging mode in the diagnostic ultrasound machine, high linearity is preferable because the unwanted harmonics need to be filtered out [26].

The direct current (DC) power consumption parameter was used because the preamplifier is a power-intensive electronic component when considering ultrasound receiver construction in the wireless ultrasound machine [41,42]. In addition, considering the preamplifier needs to enhance the weak signals, it needs to obtain high gain while sacrificing DC power consumption and occupied area [43,44]. For smartphone-based ultrasound systems with array transducers, area and power consumption are critical issues owing to the limited space and structures because unnecessary heat generation causes performance degradation during stable operation [45].

The input- and output-referred noises are the noise voltage and currents that generate the same output noises as the practical preamplifier generates if the ideal noise source is an input signal of the noise-free preamplifier [28]. The output-referred noise voltage of the preamplifier can be obtained by multiplying the gain and input-referred noise voltage of the preamplifier. The parameters of the input- and output-referred noise currents indicate the noise components of the preamplifier [46,47,48]—useful for demonstrating the noise contribution when amplifying weak echo signals through the preamplifier. Instead of input- or output-referred noise currents, a noise figure was used [49]. The preamplifier design is important because the gain of the first-stage amplifier contributes to the noise current in the receiver of the ultrasound system [50]. The noise figure (NF) equation is widely used in preamplifier design because it describes the noise contribution of the preamplifier [51]. As shown in (1), A_1_ needs to be as high as possible to reduce NF at the preamplifier [52].

(1) $$NF={NF}_{1}+\frac{{NF}_{1}-1}{A_{1}}+\ldots +\frac{{NF}_{n}-1}{A_{1}\ldots A_{n-1}},$$

where NF_1_ and NF_n_ are the noise figures of the first- and n-stage preamplifiers, respectively; A_1_ and A_n−1_ are the gains of the first- and n − 1-stage preamplifiers, respectively.

The following section presents a detailed schematic of the preamplifiers used in previously published articles on ultrasound applications.

## 3. Design Analysis of the Preamplifiers for Ultrasound Transducers

This section describes the design and schematic analysis of the design parameters of preamplifiers for specific ultrasound transducers, such as CMUT, piezoelectric transducer, and imaging. The labels and symbols in the articles are sometimes different from those in the selected articles; therefore, all schematic diagrams of the preamplifiers in this review paper were re-labeled and re-sketched, with some of the preamplifier designs also simplified to understand the operating mechanism more clearly for academic ultrasound researchers or design engineers. In the following sections, the same labels are used for input and output. B, N, and P indicate the Bipolar, N-channel metal-oxide semiconductor (NMOS), and P-channel metal-oxide-semiconductor (PMOS) transistors, respectively, while R, C, and I represent the resistor and capacitor, respectively.

### 3.1. Preamplifiers for CMUT Applications

**Figure 3:**
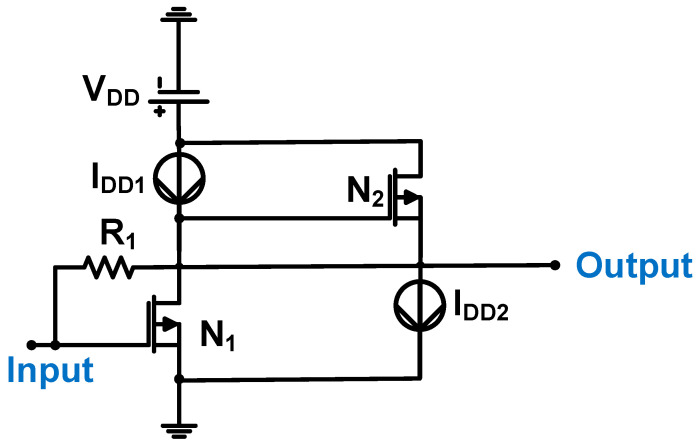
Preamplifier for CMUT device. Adapted with permission from Ref. [53]. Copyright 2009, IEEE.

Figure 3 shows a schematic of the CMUT device preamplifier. The preamplifier was constructed using a common-source amplifier (N_1_ and I_DD1_), followed by a source follower (N_2_ and I_DD2_) with a feedback resistor (R_1_). The measured gain and bandwidth of the preamplifier were 215 kΩ and 25 MHz, respectively [53].

**Figure 4:**
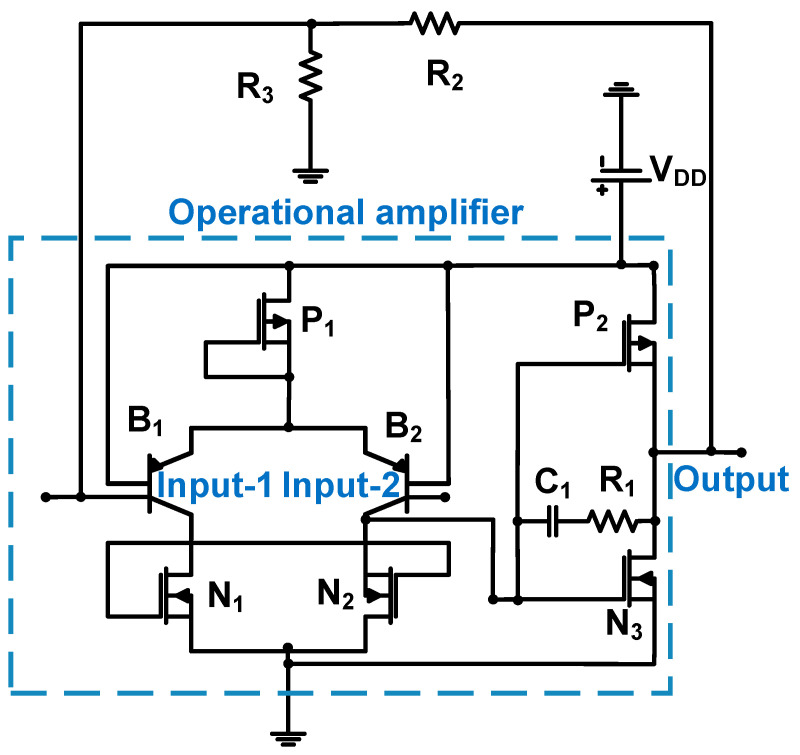
Operational amplifier with a resistor feedback loop for the CMUT device. Adapted with permission from Ref. [54]. Copyright 2005, IEEE.

Figure 4 shows a schematic of the operational amplifier with resistor feedback loops (R_2_ and R_3_) of the CMUT device. The 0.8-μm CMOS process was used; thus, the DC supply voltage is 5 V (V_DD_) [54]. This operational amplifier comprises two stages. In the first stage, a differential cascade amplifier (B_1_, B_2_, N_1_, N_2_, and P_1_) is used. In the second stage, a source follower (P_2_ or N_3_) was used to reduce the output impedance of the amplifier. A resistor (R_1_) and a capacitor (C_1_) were used to reduce the phase shift of the frequency response [55]. The measured bandwidth, DC power consumption, and input noise voltage were 11 MHz, 2 mW, and 6.45 nV/√Hz, respectively [54].

**Figure 5:**
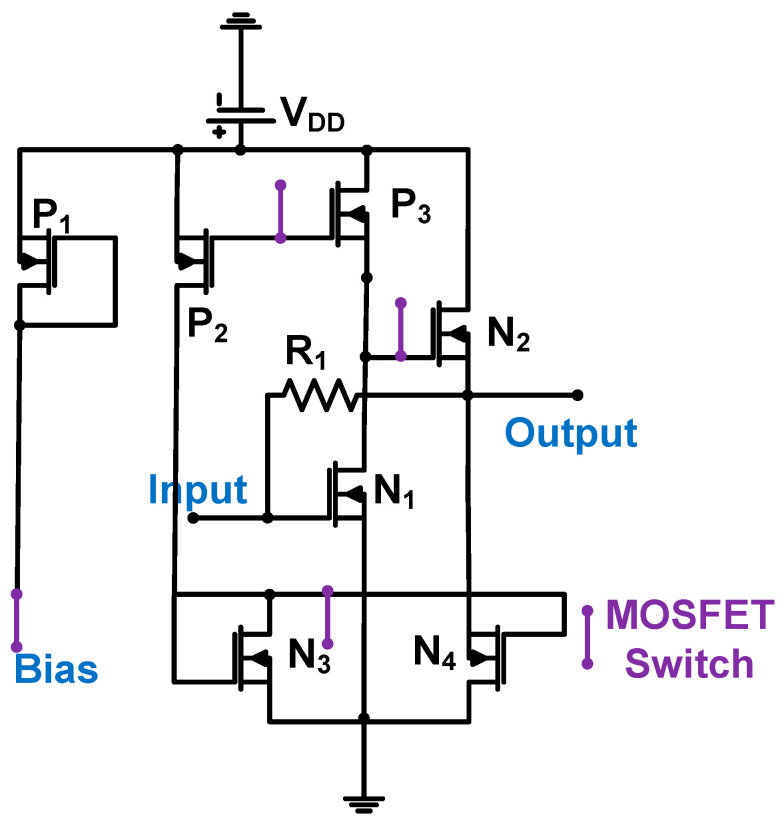
Common-source amplifier followed by a source follower with resistor feedback for the CMUT device. Adapted with permission from Ref. [56]. Copyright 2008, IEEE.

Figure 5 shows the schematic of the common-source amplifier followed by the source follower with resistor feedback for CMUT array transducer applications. The 1.5-μm CMOS process was used; thus, the DC power supply is 5 V [56]. MOSFET switches are used to turn off the power [57]. The amplifier comprises a common-source amplifier (N_1_ and P_3_), followed by a source follower (N_2_ and N_4_). A source follower was used to reduce the impedance, thus increasing the amplifier bandwidth [57], which can be expressed by Equation (2).

(2) $$Bandwidth=\frac{1}{2\pi R_{1}C_{1}},$$

where C_1_ is the feedback loop capacitance combined with the input parasitic capacitance.

The amplifier gain depends on the feedback resistance. The input-referred noise is inversely proportional to the feedback resistance (R_1_); therefore, a large R_1_ value is preferable [56]. However, the bandwidth is reduced. The bandwidth can be increased by decreasing the feedback resistance (R_1_) and feedback loop capacitance combined with the input parasitic capacitance (C_1_) [56]. However, the input-referred noise current is proportional to the √4kT/R_1_; thus, a relatively large feedback resistor is desirable if the input-referred noise current is an important design parameter [58]. The measured gain, input-referred noise current, bandwidth, and DC power consumption were 4.3 kΩ, 1.2 to 2.1 mPa/√Hz, 10 MHz, and 4 mW, respectively [56].

**Figure 6:**
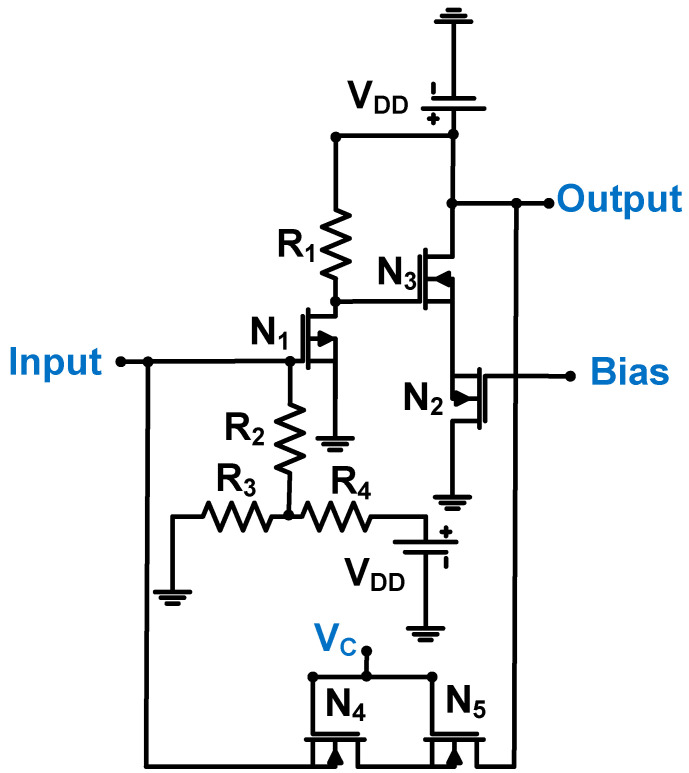
Common-source amplifier followed by a source follower with a transistor feedback loop for the CMUT device (biasing circuits are not shown for simplified analysis). Adapted with permission from Ref. [59]. Copyright 2014, IEEE.

Figure 6 shows a schematic of the common-source amplifier (N_1_), followed by a source follower (N_2_ and N_3_) with a transistor feedback loop (N_4_ and N_5_) for the CMUT device. The 0.18-μm CMOS process was used [59]. The source-connected NMOS transistors (N_4_ and N_5_) were used for the transistor feedback loop to function as resistances controlled by the DC voltage (V_c_). This topology is useful for reducing the chip area because physical resistors require large chip space [60,61,62]. The measured transimpedance gain, bandwidth, and input-referred noise are 951 dBΩ, 12 MHz, and 3.5 pA/√Hz, respectively [59].

**Figure 7:**
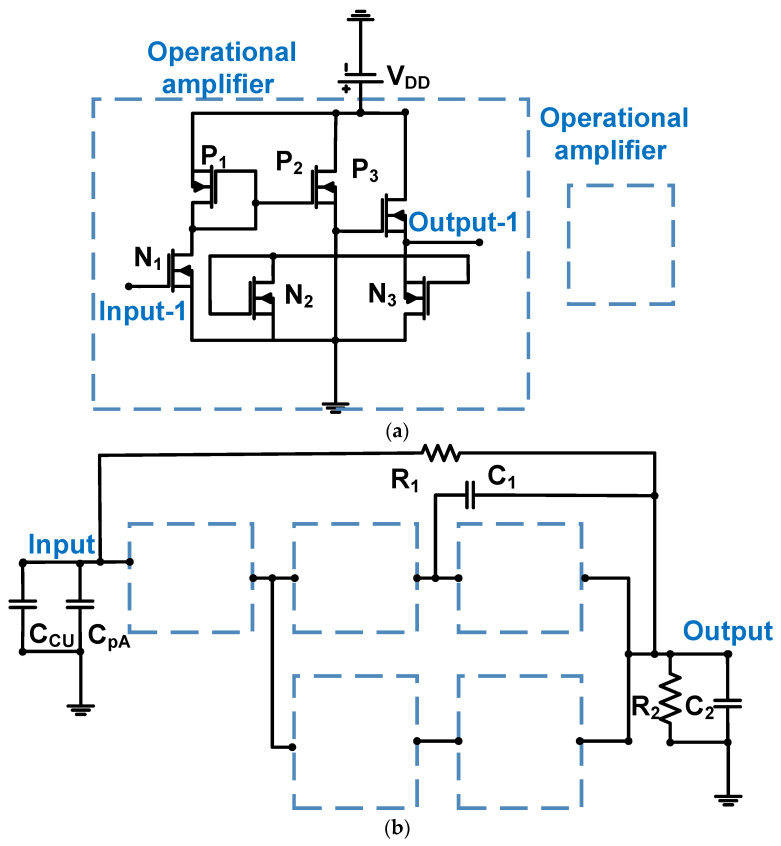
(**a**) Operational amplifier and (**b**) operational amplifier with a resistor feedback loop for CMUT. Adapted with permission from Ref. [63]. Copyright 2016, IEEE.

Figure 7a,b show the schematics of the operational amplifier with a resistor feedback loop for CMUT device applications. The 0.18-μm CMOS process was used [63]. The preamplifier was constructed using five operational amplifiers with a feedback resistor (R_1_) and a Miller capacitor (C_1_), as shown in Figure 7b. The Miller capacitor compensates for the pole and zero in the frequency response [64,65]. A current mirror (P_1_, P_2_, and P_3_) was used to reduce the power supply noise [66]. The output nodes (R_2_ and C_2_) are the input resistance and capacitance of the next stage of the electronics (ADC), respectively [63].

The input-referred current noise of the operational amplifier with resistor and capacitor feedback loop can be expressed in Equation (3) [63].

(3) $$\tilde{{i_{output}}^{2}}={\left(\frac{\tilde{V_{output-1}}}{R_{CU}∕∕R_{1}}\right)}^{2}+\omega^{2}{C_{input}}^{2}\tilde{V_{output-1}}+\frac{4kT}{R_{1}}+\frac{4kT}{R_{CU}},$$

where R_CU_ is the equivalent resistance of the CMUT and C_input_ is the combined equivalent capacitance of the CMUT and the input parasitic capacitance at the input port.

The input-referred current noise is inversely proportional to the feedback resistance (R_1_) and input capacitance (C_input_). The measured bandwidth, DC power consumption, and input-referred noise current were 4.5 MHz, 370 μW, and 1.5524 pA/√Hz, respectively [63].

**Figure 8:**
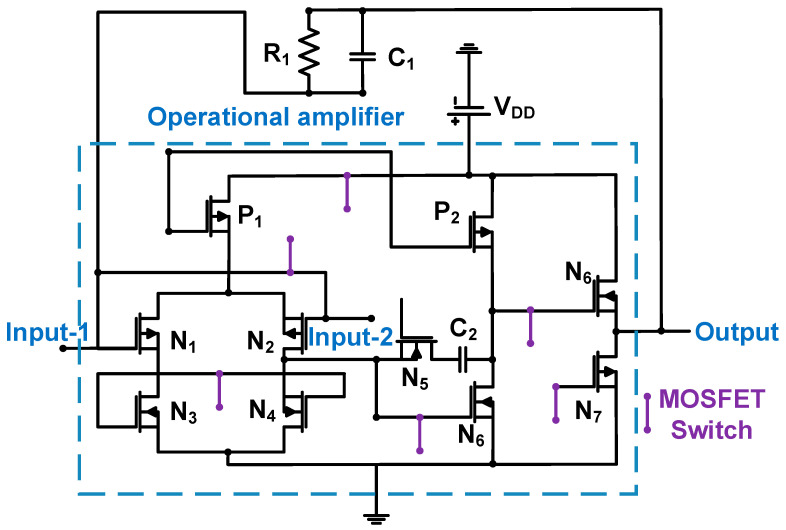
Feedback-resistor-based operational amplifier for CMUT device. Adapted with permission from Ref. [67]. Copyright 2013, IEEE.

Figure 8 shows a schematic of the operational amplifier with a feedback loop (R_1_ and C_1_). The 0.18-μm CMOS process was used [67].

Several MOSFET switches were used to reduce DC power consumption if needed. Therefore, the active DC power consumption is 14.3 mW, whereas the inactive DC power consumption is 1.5 mW [67]. The transimpedance gain of the preamplifier (A_Z_) is expressed by Equation (4) [67].

(4) $$A_{z}=\frac{R_{f}}{1+j2\pi fR_{f}C_{f}}\cdot \frac{\left(\frac{Z_{input}}{Z_{input}+Z_{feedback}}\right)A}{1+\left(\frac{Z_{i}}{Z_{input}+Z_{feedback}}\right)A},$$

where Z_input_ and Z_feedback_ are the input and feedback loop impedances, respectively; f and A are the operating frequency and open-loop gain of the operational amplifier, respectively.

The width of the NMOS (N_1_ and N_2_ = 2.3 mm) was sufficiently large to obtain a high current in the biasing circuit [67]. Different pairs and cascade stages were used to boost the gain and reduce the power supply noise, respectively, to achieve the high transimpedance gain (96.6 dBΩ) [67]. The Miller compensation capacitance (C_2_ = 5.4 pF) was used to increase the bandwidth; thus, the measured −3 dB bandwidth was 5.2 MHz [67]. The source follower (N_6_ = 135 μm/0.18 μm and N_7_ = 50 μm/0.63 μm) was used to reduce the output impedance, thus reducing the signal reflection to the next-stage component [67].

If the open loop gain of the amplifier (A) is large, the gain of the operational amplifier with feedback loop is dependent on the values of the resistance (R_1_ = 76 kΩ) and capacitance (C_1_ = 0.45 pF). The NF of the operational amplifier with feedback can be expressed by Equation (5) [67].

(5) $$NF=1+\frac{R_{input}}{R_{1}}+\frac{{\tilde{V_{output}}}^{2}}{{\tilde{I_{input}}}^{2}\left|Z_{input}\right|\cdot {\left|Z_{feedback}\right|}^{2}}+\frac{{\tilde{I_{output}}}^{2}}{{\tilde{I_{input}}}^{2}}+\frac{2\left|\tilde{V_{output}\cdot }\tilde{I_{output}}\right|}{{\tilde{I_{input}}}^{2}\cdot \left|Z_{input}\right|\cdot \left|Z_{feedback}\right|},$$

where R_input_ and R_1_ are the input and feedback loop resistances, respectively; $\tilde{I_{input}}$, $\tilde{I_{output}}$, and $\tilde{V_{input}}$ are the input, output, and input voltage currents, respectively.

In Equation (5), a large feedback loop resistance (R_1_) is desirable to reduce the NF value. The measured NF of the operational amplifier with a feedback loop was 10.3 dB at 3 MHz [67].

**Figure 9:**
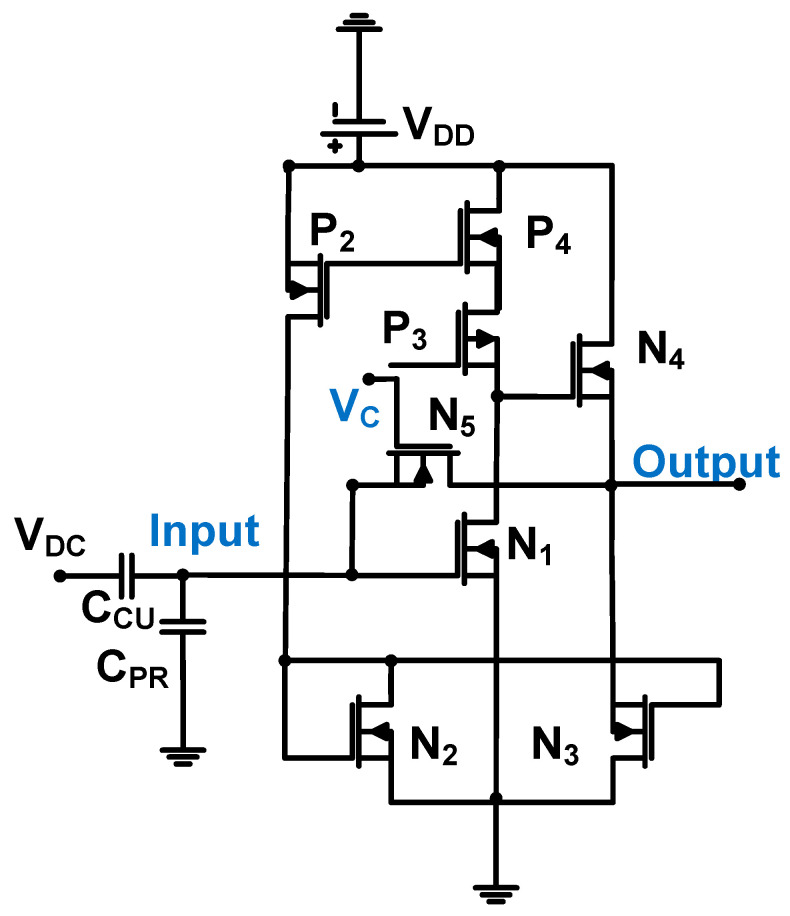
Operational amplifier with resistor feedback for CMUT device. Adapted with permission from Ref. [68]. Copyright 2011, IEEE.

Figure 9 shows a schematic of the operational amplifier with a voltage-controlled resistance (N_5_) for CMUT device applications.

Voltage-controlled resistance was implemented using the NMOS transistor to save space [69,70]. The resistance can be expressed using Equation (6) [68].

(6) $$R_{N5}=\frac{1}{\mu_{N}C_{OX}\frac{W}{L}(V_{C}-V_{OUTPUT}-V_{TH})},$$

where μ_N_ is the carrier mobility, C_ox_ is the unit-area gate capacitance, W and L are the channel width and length of the transistor, respectively, V_C_ is the bias voltage, V_OUPUT_ is the output voltage, and V_TH_ is the threshold voltage of the transistor.

The input-referred current noise of the amplifier can be expressed by Equation (7) [68].

(7) $$\tilde{{i_{in}}^{2}}=\omega^{2}{\left(C_{in}∕∕C_{CU}∕∕C_{PR}\right)}^{2}{\left(\frac{\tilde{i_{d}}}{g_{m}}\right)}^{2}+\frac{{\left(\frac{\tilde{i_{d}}}{g_{m}}\right)}^{2}}{\left(R_{CU}∕∕R_{N5}\right)}+\frac{4kT}{R_{N5}}+\tilde{{i_{CU}}^{2}},$$

where C_in_ and C_PR_ are the input and parasitic interconnect capacitances of the amplifier, respectively; C_CU_ and R_CU_ are the CMUT equivalent circuit capacitance and resistance, respectively; g_m_ is the transconductance; T is room temperature; and i_d_ and i_CU_ are the spectral densities of the current noise squares of the operational amplifier transistors and CMUT, respectively.

The input-referred current noise of the preamplifier is proportional to the input and parasitic interconnect capacitances of the preamplifier and the CMUT equivalent circuit capacitance but is inversely proportional to the voltage-controlled resistance [68].

The transimpedance gain of the preamplifier (A_Z_) is expressed in Equation (8) [68].

(8) $$A_{z}=\frac{R_{N5}{\omega_{0}}^{2}}{s^{2}+\frac{\omega_{0}}{Q}s+{\omega_{0}}^{2}},$$

where ω_0_ and Q are the radian bandwidth and quality factor of the amplifier.

As shown in Equations (7) and (8), a high resistance (R_N5_) can lower the input-referred current noise and increase the transimpedance gain of the preamplifier. The measured DC power consumption, transimpedance gain, bandwidth, and input current noise density were 6.6 mW, 3 MΩ, 20 MHz, and 90 fA/√Hz, respectively [68].

**Figure 10:**
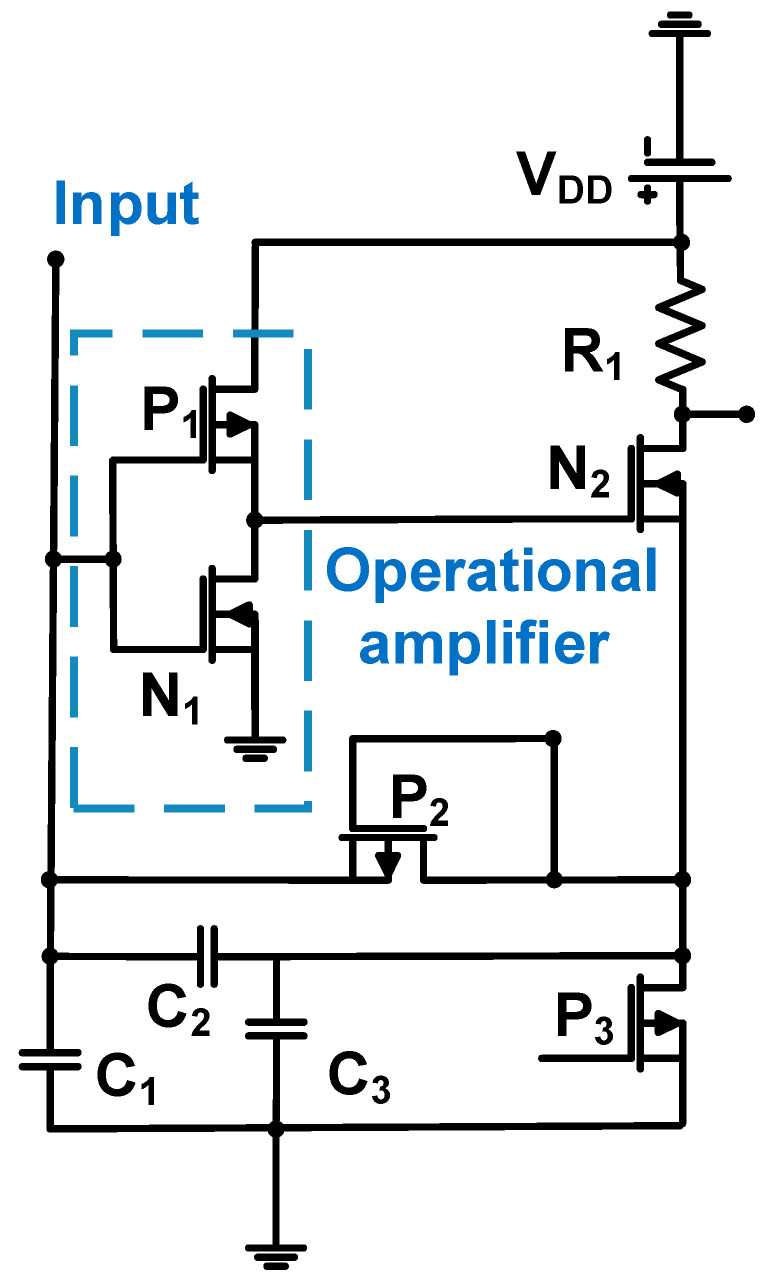
Feedback-capacitor-based operational amplifier for CMUT devices used in the IVUS and ICE device applications. Adapted with permission from Ref. [71]. Copyright 2014, IEEE.

Figure 10 shows a schematic of the two-stage operational amplifier with a capacitive feedback loop (C_2_ and C_3_) for the CMUT applications. The 0.35-μm CMOS process was used; thus, the DC power supply (V_DD_) is 3.3 V [71]. In the first stage, an operational amplifier was constructed using NMOS (N_1_) and PMOS (P_1_) transistors. In the second stage, the source follower was constructed using NMOS (N_2_) and PMOS (P_3_) transistors.

The operational amplifier comprises a capacitor feedback loop (C_2_ and C_3_). Therefore, the transfer function of the amplifier with a capacitor feedback loop (I_Z_) is expressed as Equation (9) [71].

(9) $$I_{z}\left(s\right)={\left[\frac{s^{2}}{\frac{C_{2}A\omega_{0}g_{N2}}{C_{3}\left(C_{1}+C_{2}\right)\left(\omega_{0}+\frac{g_{N2}}{C_{3}}\right)}}+\frac{s}{\frac{C_{2}A\omega_{0}g_{N2}}{C_{3}\left(C_{1}+C_{2}\right)}+1}\right]}^{-1},$$

where s is the complex operating frequency, ω_0_ is the radian bandwidth of the operational amplifier, g_N2_ is the transconductance of the MOSFET of N_2_, A is the open-loop voltage gain, and C_1_ is the combined capacitances of the CMUT and parasitic interconnection.

The gain of the operational amplifier with a capacitor feedback loop can be expressed by Equation (10) [71].

(10) $$A_{z}\left(s\right)=\frac{\left(1+\frac{C_{2}}{C_{1}})R_{1}(1+\frac{s}{\frac{C_{2}A\omega_{0}}{C_{1}}}\right)}{1+\frac{s}{\frac{\frac{C_{1}A\omega_{0}g_{N2}}{C_{3}\left(C_{1}+C_{2}\right)}}{\omega_{0}+\frac{g_{N2}}{C_{3}}}}+\frac{s^{2}}{\frac{C_{1}A\omega_{0}g_{N2}}{C_{3}\left(C_{1}+C_{2}\right)}}}$$

The measured −3 dB bandwidth, transimpedance gain, and DC power consumption were 40 MHz, 200 kΩ, and 0.8 mW, respectively. The input-referred spectral density of the amplifier current noise is expressed as Equation (11) [71].

(11) $$\tilde{{I_{input}}^{2}}=\frac{1}{{\left(1+\frac{C_{3}}{C_{2}}\right)}^{2}}\left(\frac{4kT}{R_{1}}+{i_{db}}^{2}\right)+\omega^{2}{\left(C_{1}+C_{2}\right)}^{2}\frac{\tilde{i_{d}}}{g_{N2}},$$

where k is a process-dependent constant, g_m_ is the MOSFET transconductance, i_d_ is the spectral density of the current noise square of the operational amplifier transistors, and i_db_ is the spectral density of the current noise square of the current-bias circuit.

The transconductance (g_N2_) and load resistance (R_1_) must be high to reduce the input-referred spectral density of the amplifier current noise. The measured input referred noise at 20 MHz was 0.31 pA/√Hz [71].

### 3.2. Preamplifiers for Piezoelectric Transducer Applications

**Figure 11:**
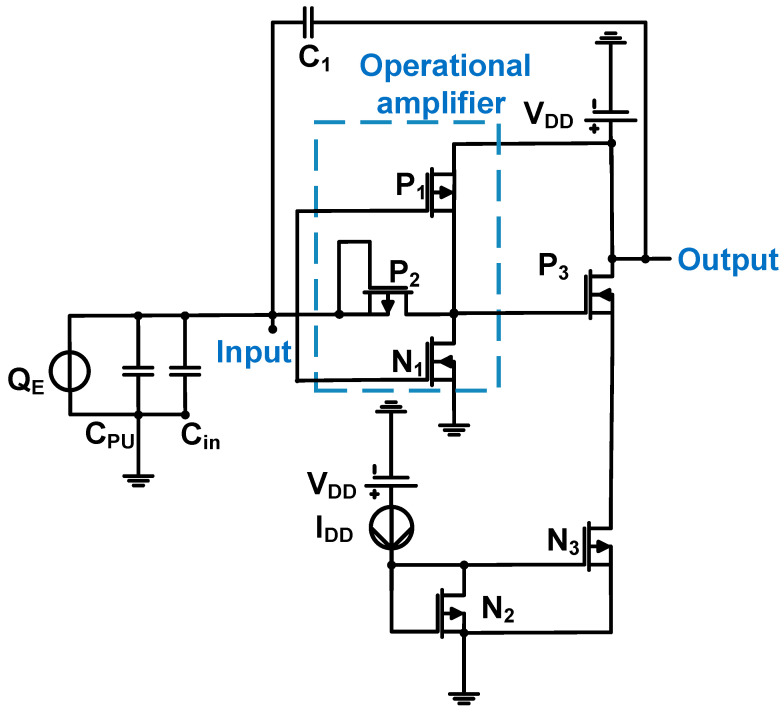
Operational amplifier with a capacitor feedback loop for the PMUT device. Adapted from Zamora et al. [72] with permission under the terms of the CC BY 4.0 License, Copyright 2020 MDPI AG.

Figure 11 shows a schematic of the operational amplifier, followed by a source follower with a capacitor feedback loop for piezoelectric micromachined ultrasonic transducer (PMUT) array applications. The source follower is constructed using NMOS (N_3_) and PMOS (P_3_). The 0.13-μm CMOS process was used [72]. In the first stage, an operational amplifier was constructed using NMOS (N_1_) and PMOS (P_1_) transistors. In the second stage, a source follower was constructed using NMOS (N_3_) and PMOS (P_3_) transistors.

The output of the operational amplifier with a capacitive feedback loop (C_1_) can be simplified using Equation (12) if the open-loop gain of the amplifier is high [72].

(12) $$V_{out}=Q_{E}/C_{1},$$

where Q_E_ is the electric charge produced by the PMUT device and C_1_ is the feedback loop capacitance.

The input-referred current noise of the amplifier is proportional to the input capacitance of the operational amplifier (C_in_) and feedback capacitance (C_1_); thus, it can be expressed using Equation (13) [72].

(13) $$\tilde{i_{in}(s)}=\frac{sC_{in}(C_{1}+C_{in})(i_{nn}+i_{np})}{\left(g_{N1}+g_{P1}\right)(1-\frac{sC_{1}}{g_{N1}+g_{P1}})},$$

where C_in_ is the electric charge produced by the PMUT device, g_N1,_ and g_P1_ are the transconductances of MOSFET N_1_ and P_1_, respectively, and i_nn_ and i_np_ are the square root mean square current noises of MOSFET N_1_ and P_1_, respectively.

The voltage gain, bandwidth, DC power consumption, and input referred noise of the preamplifier were 21.8 dB, 22 MHz, 0.3 mW, and 7.1 nV/√Hz at 3 MHz, respectively [72].

**Figure 12:**
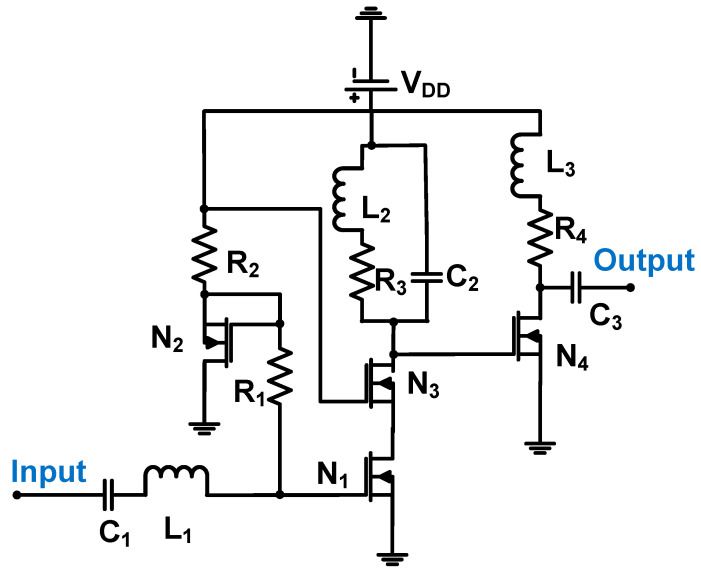
LNA for piezoelectric transducer devices (electrostatic discharge device (ESD) and electrostatic capacitors not shown for simplified analysis). Adapted with permission with Ref. [73]. Copyright 2011, IEEE.

Figure 12 shows a schematic of the low-noise amplifier (LNA) used for high-frequency piezoelectric transducer applications. The 0.18-μm BiCMOS process was used [73]. The LNA was constructed using a cascade amplifier (N_1_ and N_3_), followed by a common-source amplifier (N_4_) with a resonant load (R_3_, C_2_, L_2_, L_3_, and R_4_) owing to its high-frequency piezoelectric transducer characteristics [73].

The voltage gain of the amplifier can be expressed as Equation (14) [73]:

(14) $$A_{V}=\frac{g_{N1}g_{N3}C_{1}}{C_{ESD}+C_{gsN1}}\frac{g_{N4}\sqrt{{R_{4}}^{2}+{\left(\omega L_{3}\right)}^{2}}}{\omega C_{2}+\frac{1}{\sqrt{{R_{3}}^{2}+{\left(\omega L_{2}\right)}^{2}}}},$$

where g_N1_, g_N3_, and g_N4_ are the transconductances of MOSFET N_1_, N_3_, and N_4_, respectively, and C_ESD_ and C_gsN1_ are the ESD and gate-source parasitic capacitances of the MOSFET N_1_.

The voltage gain of the amplifier can be related to the load impedances (R_3_, R_4_, L_2_, L_3_, and C_2_), transconductance (g_N1_, g_N3_, and g_N4_), ESD parasitic capacitance, and gate-source parasitic capacitance of MOSFET N_1_. The measured voltage gain, bandwidth, and DC power consumption of LNA were 24.08, 73, and 43.57 mW, respectively [73].

The noise figure (NF) of the preamplifier can be expressed using Equation (15) [73].

(15) $$NF=1+\frac{r_{N1}+\frac{1}{2g_{N1}}}{\sqrt{{\left(\frac{1}{\omega C_{1}}\right)}^{2}+{\left(\omega L_{1}\right)}^{2}+{\left(\frac{1}{\omega {(C}_{ESD}+C_{gsN1})}\right)}^{2}}},$$

where r_N1_ gate resistance of the MOSFET N_1_.

The NF of the LNA can be improved by a large transconductance (g_N1_) and low input, ESD parasitic capacitance, and gate-source parasitic capacitance of MOSFET N_1_ (C_1_, C_ESD_, and C_GSN1_). The measured NF of the amplifier is 3.51 dB [73].

**Figure 13:**
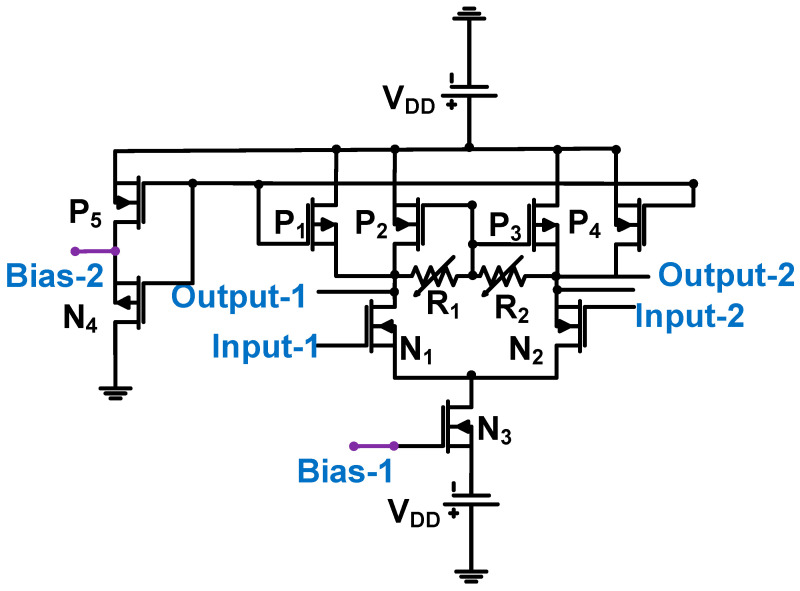
Preamplifier for piezoelectric transducer device (biasing circuits not shown for simplified analysis) [74]. Copyright 2009, IEEE.

Figure 13 shows a schematic of the preamplifier used in piezoelectric transducer applications. The acoustic signals from the ultrasound transducer were sent to the Input-1 port, with the common ground of the transducer connected to the Input-2 port [74]. The voltage gain depends on the variable resistors (R_1_ and R_2_) and the transistor sizes (N_1_, P_2_, N_2_, and P_3_). The measured gain, bandwidth, and NF of the preamplifier were 20, 75, and 10 dB, respectively [74].

**Figure 14:**
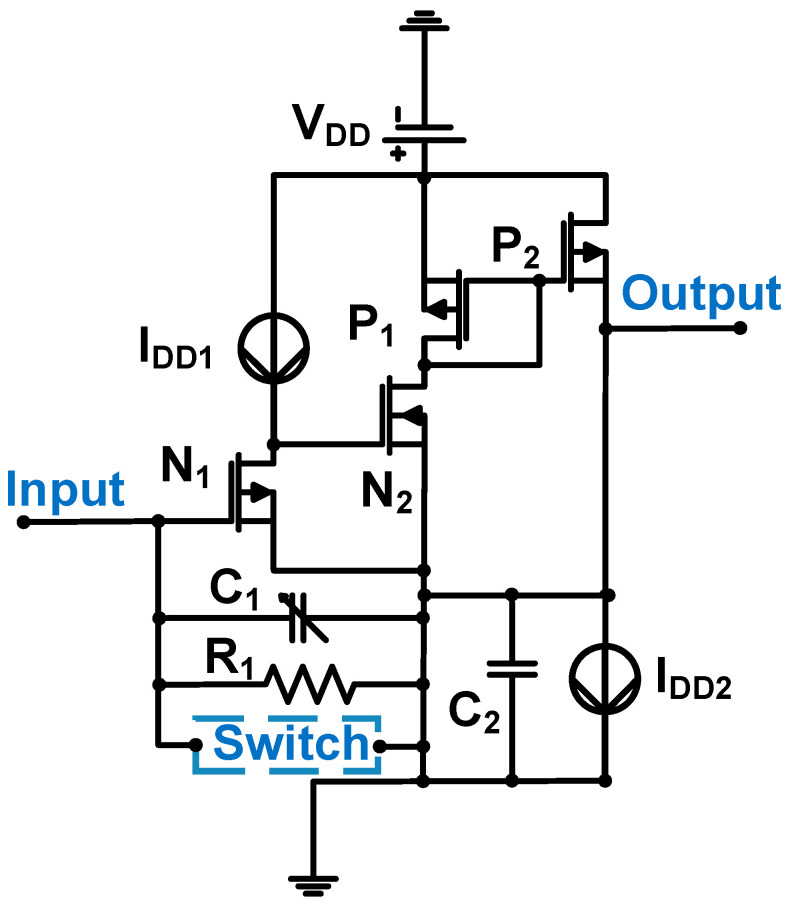
LNA for piezoelectric transducer device (switch circuits not shown because they are not related to analysis). Adapted with permission from Ref. [75]. Copyright 2021, IEEE.

Figure 14 shows a schematic of an LNA. The 0.18-μm CMOS process was used; thus, the DC supply voltage (V_DD_) is 3 V [75].

The LNA is constructed using a three-stage common-source amplifier. The transistors (N_1_ and P_2_) were biased to obtain the 600 μA current (I_DD1_) and 800 μA current (I_DD2_), respectively [75]. Resistor (R_1_) can prevent leakage currents for long-cycle pulse signals and capacitor C_1_ can be programmed with 6-dB steps [75]. Therefore, the gain of the LNA (A_I_) can be expressed by Equation (16) [75].

(16) $$A_{I}=1+\frac{C_{2}}{C_{1}}$$

The measured center frequency, bandwidth, and input-referred noise current were 13 MHz, 21 MHz, and 4 nA/√Hz [75].

**Figure 15:**
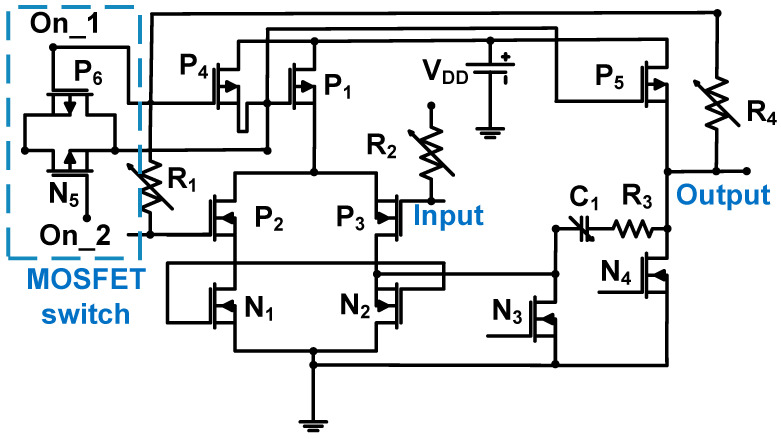
Variable LNA for piezoelectric transducer device. Adapted with permission from Ref. [77]. Copyright 2018, IEEE.

Figure 15 shows a schematic of the variable LNA with a resistor feedback loop for the piezoelectric transducer because the LNA topology is preferable for low impedance [76]. A variable LNA with a resistor feedback loop was used because of the signal attenuation of echo signals in deep areas [77]. The 0.18-μm CMOS process was used [77]. This two-stage variable LNA structure had a feedback loop composed of two variable resistors (R_1_ and R_4_). The first stage of the LNA is a cascade amplifier composed of an NMOS (N_1_ and N_2_) and PMOS (P_1_, P_2_, and P_3_), and the second stage is the source follower (N_4_ and P_5_). A variable Miller capacitor (C_1_) is used to increase the bandwidth by improving the phase margins [78]. A MOSFET switch composed of transistors (N_5_ and P_6_) was used to reduce the power consumption of the LNA during the period when the driving pulse signals were applied.

The voltage gain of the LNA can be expressed as Equation (17) [77]:

(17) $$A_{V}=1+\frac{R_{4}}{R_{1}}$$

The measured gain, bandwidth, and input-referred noise voltage of the variable LNA with resistor feedback loop were 32 dB, 11 MHz, and 4.1 nV/√Hz, respectively [77].

### 3.3. Preamplifier for Ultrasound Imaging Applications

**Figure 16:**
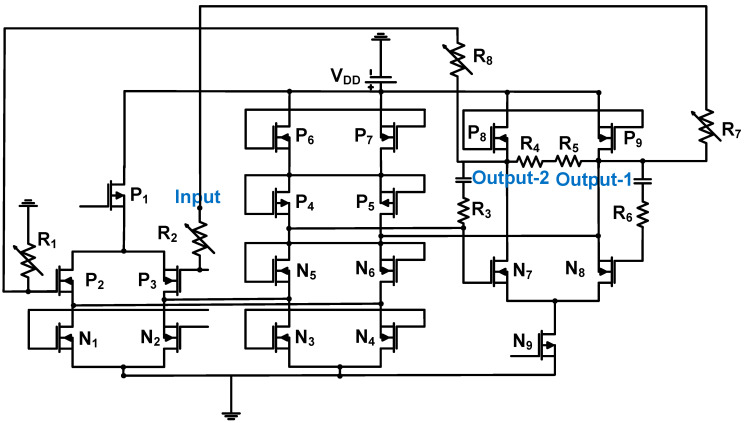
LNA for 3-D ultrasound imaging applications. Adapted with permission Ref. [79]. Copyright 2018, IEEE.

Figure 16 shows a schematic of the LNA for ultrasound imaging applications. The 0.18-μm CMOS process was used [79]. The LNA was constructed using a three-stage operational amplifier with feedback resistors (R_7_ and R_8_) and variable input resistors (R_1_ and R_2_ = 0.2, 0.4, 0.8, and 1.6 kΩ) [79]. The PMOS inputs were used to reduce the noise of the preamplifier. The gain of the LNA was dependent on the variable resistors (R_1_, R_2_, R_7_, and R_8_). The measured gain, bandwidth, OIP_3_, and input-referred noise current of the LNA were 15.6 dB, 10 MHz, 2.64 Vp-p, and 6.3 nV/√Hz, respectively [79].

## 4. Discussion and Conclusions

This review will guide the design characteristics of preamplifiers for ultrasound transducer applications. For ultrasound applications, currently used most IC fabrication processes are 0.13 μm, 0.18 μm, or 0.8 μm because the supply voltage of the 0.13 μm, 0.18 μm, and 0.8 μm IC fabrication processes are 1.8 V, 3.3 V, and 5 V, respectively. Below the 0.13-μm process, the supply voltage is lower than 1.8 V; as a result, the maximum achievable gain of the preamplifier could be limited even though high dynamic ranges of the preamplifier are desirable. Therefore, a new IC fabrication process may not be desirable even if the sizes of the new IC fabrication processes are smaller.

The primary design parameters of the preamplifier are gain, bandwidth, noise figure (or input- or output-referred noise), power consumption, and IIP_3_ or OIP_3_ [80,81]. These design parameters of the preamplifiers have a trade-off relationship; therefore, circuit or system designers must consider the parameter specifications for the performance of ultrasound transducers. For example, the bandwidth of a preamplifier should be larger than that of an ultrasound transducer. The input-referred noise of the preamplifier must be similar to or lower than that of the ultrasound transducer. The gain of the preamplifier should be high if the sensitivity of the transducer used in the IVUS applications is low. However, the bandwidth of the preamplifier for the operational amplifier type can be increased if its gain of the preamplifier needs is decreased [82]. While a high biasing current can increase the gain of the preamplifier, it causes unnecessary DC power consumption; therefore, an appropriate current is desirable at the design level [83]. A preamplifier with a wide bandwidth can increase the number of unwanted harmonic components of the acoustic signals generated by the ultrasound transducer. While a high linearity of the preamplifier can be obtained if a current-biasing circuit based on the MOSFET is used, it causes high DC power consumption [84].

For CMUT device applications, a transimpedance amplifier—an operational amplifier with a feedback loop composed of resistors or capacitors—is preferred for high impedances [85,86]. For piezoelectric devices, the LNA is preferable because of the low impedance of the piezoelectric transducer [72].

To increase the gain of the preamplifier, circuit designers use a common-source amplifier with a large width of the first transistor connected to the input port or use a cascade topology to obtain a high current from the biasing circuit [28]. However, this causes relatively high DC power consumption. In the last stage, the source follower is used to reduce the output impedance, thus smoothly passing the amplified signal to the next-stage amplifier or ADC. In an operational amplifier with a resistor feedback loop, the feedback resistor affects the gain and bandwidth of the preamplifier.

For the input-referred noise or NF, the transconductance value of the MOSFET is important because it can affect the noise of the preamplifier [87]. For an operational amplifier with a feedback resistor loop, the feedback resistor can affect the noise parameters [66]. In addition, the open-loop gain of the operational amplifier must be large to reduce the input-referred noise [28]. MOSFET switches can be used to reduce power consumption during the driving pulse period when transmitted signals are applied [88]. Instead of resistors, voltage-controlled MOSFETs for amplifier design could help reduce the chip area [88]. However, this scheme may require an integrated preamplifier design with a more complex and accurate timing period after pulse transmission. In particular, this technique can help reduce power consumption in wireless ultrasound systems. Miller capacitors in the output port are sometimes used to increase the bandwidth by moving the pole and zero locations [30]. An operational amplifier with a capacitive feedback loop was used for the PUMT device, which has a lower impedance than that of the CMUT [72]. An LNA with a resonant load was developed for high-frequency piezoelectric transducers [73]. The LNA constructed using a three-stage common-source amplifier used resistors to prevent leakage currents for a long-cycle transmission period and a capacitor to provide a 6-dB step gain [77].

Table 1 summarizes the design parameters of the previously published preamplifiers used for ultrasound transducers. As shown in Table 1, the gain of the transimpedance amplifier is expressed by dBΩ or kΩ units because the input is current and the output is voltage. The input-referred noise (IRN) can be expressed by current or voltage units, and the NF can be expressed on the dB scale. Topologies can be classified into common source, operational amplifier, or low-noise amplifier types.

While several review papers on IC components for ultrasound systems have been published, they did not provide specific design guidelines for preamplifiers used in ultrasound transducer applications. Therefore, this is the first review paper of preamplifiers to provide design guidelines for ultrasound transducer applications, such as capacitive micromachined ultrasonic transducer (CMUT), piezoelectric transducer, and ultrasound imaging applications.

In ultrasound imaging, preamplifiers are required to amplify weak acoustic signals and obtain images for diagnostic purposes. However, their performance is limited because of transistor requirements. Therefore, the design parameters of the gain, bandwidth, input- or output-referred noise currents, and DC power consumption are described to explain the design concepts of the preamplifiers because they have a trade-off relationship when designing the preamplifier components used for diagnostic ultrasound imaging applications

Recently, with the emergence of new ultrasound applications such as photoacoustic imaging, smartphone touch sensors, wireless ultrasound machines, brain stimulation, and ultrasound-combined positron emission tomography, academic researchers have used commercial components or system IC for these emerging applications. However, further performance optimization is possible if ultrasound transducers with appropriate electronic selection or a design topology that considers a trade-off relationship are developed. As such, the knowledge of preamplifier design in this review paper is expected to be helpful in this regard.

## Figures and Tables

**Table 1:** Summary of the preamplifiers currently used for ultrasound transducer research.

Paper	Gain	Bandwidth	DC Power Consumption	IIP_3_ or OIP_3‘_	IRN	NF	Topology	Application
[53]	215 kΩ	25 MHz	–	–	–	–	Common-source amplifier and source follower with a feedback resistor	CMUT
[54]	–	11 MHz	2 mW	–	6.45 nV/√Hz	–	Operational amplifier with resistor feedback	CMUT
[56]	4.3 kΩ	10 MHz	4 mW	–	2.1 mPa/√Hz	–	Common-source amplifier and source follower with a feedback resistor	CMUT
[59]	95.1 dBΩ	12 MHz	–	–	3.5 pA/√Hz	–	Common-source amplifier and source follower with a transistor feedback loop	CMUT
[63]	–	4.5 MHz	0.37 mW	–	1.5524 pA/√Hz	–	Operational amplifier with resistor feedback	CMUT
[67]	96.6 dBΩ	5.2 MHz	14.3 mW	618 mV (OIP_3_)	–	10.3 dB	Operational amplifier with a feedback loop	CMUT
[68]	3 MΩ	20 MHz	6.6 mW	–	90 fA/√Hz	–	Operational amplifier with resistor feedback	CMUT
[71]	200 kΩ	40 MHz	0.8 mW	–	0.31 pA/√Hz	–	Operational amplifier with capacitor feedback	CMUT
[72]	21.8 dB	22 MHz	0.3 mW	–	7.1 nV/√Hz	–	Operational amplifier with resistor feedback	Piezoelectric transducer
[73]	24.08 dB	73 MHz	43.57 mW	−3.5 dB_m_ (IIP_3_)	–	3.51 dB	Low-noise amplifier with a resonant circuit	Piezoelectric transducer
[74]	20 dB	75 MHz	–	–	–	10 dB	Operational amplifier with resistor feedback	Piezoelectric transducer
[75]	69 dB	21 MHz	–	–	4 nA/√Hz	–	Low-noise amplifier	Piezoelectric transducer
[77]	32 dB	11 MHz	–	–	4.1 nV/√Hz	–	Variable low-noise amplifier with resistor feedback	Piezoelectric transducer
[79]	15.6 dB	10 MHz	–	2.64 V_p-p_ (OIP_3_)	6.3 nV/√Hz	–	Low-noise amplifier	Imaging

## Data Availability

Data are contained within the article.

## Abbreviations

AC	Alternating current
DC	Direct current
ADC	Analog-to-digital converter
DAC	Digital-to-analog converter
CMUT	Capacitive micromachined ultrasonic transducer
DAC	Digital-to-analog converter
IIP_3_	Third-order input intercept point
OIP_3_	Third-order output intercept point
NMOS	N-channel MOS
PMOS	P-channel MOS
MOSFET	Metal-oxide-semiconductor field-effect transistor
THD	Total harmonic distortion
PMUT	Piezoelectric micromachined ultrasonic transducer
TIA	Transimpedance amplifier
LNA	Low-noise amplifier
VGA	Variable gain amplifier
PGA	Programmable gain amplifier
TGCA	Time gain compensation amplifier
CMOS	Complementary Metal-oxide-semiconductor
IVUS	Intravascular ultrasound system
SNR	Signal-to-noise ratio
NF	Noise figure
IC	Integrated circuit
IRN	Input-referred noise current
ASIC	Asynchronous semiconductor integrated circuit
ESD	Electrostatic discharge device
